# Glutathione peroxidase 4 (GPX4) and obesity interact to impact tumor progression and treatment response in triple negative breast cancer

**DOI:** 10.1186/s40170-025-00380-8

**Published:** 2025-02-25

**Authors:** Emily N. Devericks, Bennett H. Brosnan, Alyssa N. Ho, Elaine M. Glenny, Hannah M. Malian, Dorothy Teegarden, Michael K. Wendt, Michael F. Coleman, Stephen D. Hursting

**Affiliations:** 1 Department of Nutrition, University of North Carolina, Chapel Hill, NC USA; 2 Department of Nutrition Science, Purdue University, West Lafayette, IN USA; 3 Purdue University, Purdue University Institute for Cancer Research, West Lafayette, IN USA; 4 Holden Comprehensive Cancer Center, University of Iowa, Iowa City, IA USA; 5 Department of Internal Medicine, University of Iowa, Iowa City, IA USA; 6 Lineberger Comprehensive Cancer Center, University of North Carolina, Chapel Hill, NC USA; 7 Nutrition Research Institute, University of North Carolina, Kannapolis, NC USA

## Abstract

**Introduction:**

Triple-negative breast cancer (TNBC), which tends to be more advanced when diagnosed and more aggressive than other breast cancer subtypes, is accelerated by obesity. Hypertrophic adipocytes and cancer cells exhibit increased oxidative stress and altered redox homeostasis, influencing therapeutic outcomes. Enzymes implicated in both redox regulation and TNBC include glutathione peroxidase 4 (GPX4; reduces lipid peroxides) and pyruvate carboxylase (PC; essential in oxidative stress protection). Using preclinical models, we characterized interactions between GPX4, PC, and oxidative stress in TNBC cells, and established effects of GPX4 suppression on TNBC progression. In TNBC cells, PC knockdown increased GPX4 expression, while GPX4 knockdown increased PC expression. GPX4 inhibition by erastin or RSL3 enhanced TNBC cell death in vitro, and antioxidants mitigated the cytotoxicity. In obese mice, GPX4 knockdown, versus scramble control: (i) reduced tumor burden following orthotopic transplantation of TNBC cells; and (ii) reduced lung metastasis following tail vein injection of TNBC cells in combination with chemotherapy (carboplatin) but not immunotherapy (anti-CTLA4 plus anti-PD1). We conclude that GPX4 and PC expression are inversely related in TNBC cells, and GPX4 and obesity interact to impact TNBC progression and treatment responses. Moreover, GPX4-mediated redox defense, alone or in combination with chemotherapy, is a targetable vulnerability for treating TNBC, including obesity-related TNBC.

**Implication:**

GPX4 suppression, alone or with current TNBC therapies, impacts outcomes in preclinical TNBC models with or without obesity and offers a new, plausible mechanistic target for TNBC treatment.

**Supplementary Information:**

The online version contains supplementary material available at 10.1186/s40170-025-00380-8.

## Introduction

Breast cancer is the most common cancer in women in the US, affecting about 1 in 8 women during their lifetime [1]. Obesity occurs in more than 40% of US women [2] and is an established risk and progression factor for several breast cancer subtypes including triple-negative breast cancer (TNBC) [3]. About 10–15% of breast cancers are TNBC, which has higher metastatic potential and lower survival rates than other subtypes [4], and until recently lacked targeted therapies. Accordingly, new molecular targets and treatment strategies for TNBC, including obesity-related TNBC, are urgently needed.

Oxidative stress and inflammation share several biological signaling pathways, frequently co-occur, and have emerged as key mediators of both obesity and breast cancer [5, 6]. TNBC is particularly sensitive to lipid-specific oxidative stress [7], with lipid peroxide levels rising significantly as TNBC progresses [8]. Obesity induces various systemic metabolic changes including hyperlipidemia, hyperglycemia, oxidative stress and decreased antioxidant capacity [9, 10]. Increased levels of reactive oxygen species within the tumor microenvironment and surrounding adipose tissue is an established mechanism through which obesity drives breast cancer growth, causing accumulation of lipid and protein oxidation products [11]. Glutathione peroxidase 4 (GPX4) is a key metabolic enzyme that reduces lipid peroxide levels, inhibits ferroptosis (a form of programmed cell death characterized by lipid peroxide accumulation), and mitigates inflammation-related lipid signaling [12, 13]. While GPX4 is an important regulator of metastatic progression of breast cancer and adaptive response to lipid accumulation [14], the role of GPX4 in obesity-related TNBC progression has not yet been reported.

Reactive oxygen species (ROS) exert paradoxical effects on tumor development and survival, whereby moderate levels promote signaling pathways driving malignant transformation of healthy cells and cancer progression, and high levels promote cellular senescence and tumor cell death [15]. Pyruvate carboxylase (PC) is an anaplerotic enzyme essential for oxidative stress protection, although its relationship with GPX4 in obesity-related breast cancer is not established. Tumor cells compensate for upregulation of ROS production and signaling with increased antioxidant capabilities, promoting their survival and driving resistance to some therapies [16]. Redox-targeting treatments are gaining popularity as part of an adjuvant or neoadjuvant therapeutic strategy [17]. Tumor dependence on lipid ROS-specific antioxidants is important in several forms of therapy resistance [18], and creating a redox imbalance via glutathione depletion increases chemotherapy efficacy [19]. Platinum-based compounds such as carboplatin that are used to treat TNBC [20] directly increase lipid oxidative stress [21]. Immunotherapy treatments, such as anti-CTLA4 and anti-PD1 immune checkpoint inhibition (ICI), also elevate oxidative stress [22] and are increasingly being combined with chemotherapy for treating TNBC [23]. The role of GPX4 in the effectiveness of platinum-based chemotherapy or ICI treatment is unclear.

We and others postulate that the unique, delicately balanced redox biology of cancer cells may reveal new molecular targets for TNBC treatment [24]. Herein, using in vitro TNBC models including cell lines with GPX4 or PC suppressed, together with murine models of obesity and TNBC growth and metastasis, we aimed to characterize (i) interactions between GPX4, PC, and lipid oxidative stress in TNBC cells, and (ii) the effect of GPX4 suppression, alone or in combination with chemotherapy or immunotherapy, on TNBC progression.

## Methods

### Cell culture and in vitro TNBC models

M-Wnt and metM-Wnt^lung^ murine mammary cancer cells were derived in the Hursting laboratory [25], and E0771 cells were purchased from ATCC (#CRL-3461; Gaithersburg, MD). Cells were maintained in RPMI-1640 media supplemented with 10% fetal bovine serum (FBS), 1% penicillin–streptomycin, 2 mM L-glutamine, and 10 mM HEPES buffer. Prior to experiments, cells were seeded in human plasma-like media (HPLM) containing 10% FBS and 1% penicillin–streptomycin. All cell lines were routinely tested for mycoplasma using the Universal Mycoplasma Detection Kit (ATCC, #30–1012 K).

#### PC knockdown

PC was suppressed in metM-Wnt^lung^ cells and E0771 cells by lentiviral transduction for 48 h, using particles generated by transfection of HEK293T cells with psPAX2 (Addgene), pMD2.G (Addgene), and Pcx-targeting shRNAs. PC was suppressed in E0771 cells with TRC lentiviral mouse Pcx-targeting shRNAs (lentiviral pLKO.1 TRC cloning vector) purchased from GE Dharmacon (AAAGGACAAATAGCTGAAGGG [shPC25], TTGACCTCGATGAAGTAGTGC [shPC28], and TTCTCCGAACGTGTCACGT [Scram]) and were selected for using puromycin (5 µg/ml). Similarly, PC was suppressed in E0771 and metM-Wnt^lung^ cells with Pcx-targeting shRNA purchased from Genecopoeia in a psi-LVRU6H vector (GGAGTTGGAAGAGAATTACAC [shPC-B], CCCACAACTTCAACAAGCTCT [shPC-C], and GCTTCGCGCCGTAGTCTTA [Scram]) and were selected for using hygromycin (500 µg/ml).

#### GPX4 knockdown

GPX4 was suppressed in metM-Wnt^lung^ cells by lentiviral transduction for 48 h, using particles generated by transfection of HEK293T cells with psPAX2, pMD2.G, and GPX4-targeting shRNAs in a psi-LVRU6H vector (Genecopoeia, GCACGAATTCTCAGCCAAGGA [shGPX4-A], GCTGGGAAATGCCATCAAATG [shGPX4-C], and GCTTCGCGCCGTAGTCTTA [Scram]) and were selected for using hygromycin (500 µg/ml).

### Cell viability and proliferation assays

Parental E0771, M-Wnt, and metM-Wnt^lung^ cells, as well as GPX4- and PC-knockdown and shScramble control E0771 and metM-Wnt^lung^ cells, were seeded at 5 × 10^3^ cells/well in a 96-well plate in HPLM overnight, then treated with a ferroptosis inducer (erastin or RAS-selective lethal 3 (RSL3) [26, 27]) or carboplatin across a range of concentrations, antioxidants (1mM N-acetyl cysteine, 2.5 µM liproxstatin-1 [28], 2.5-5 µM ferrostatin-1 [26]), or a general ROS inducer H_2_O_2_ (500 µM). After 24 h of treatment, cell numbers were quantified by staining with 100 µl of MTT solution (0.5 mg/mL 3-(4,5-dimethylthiazol-2-yl)-2,5-diphenyl tetrazolium bromide in HPLM without FBS) for 90 min, then solubilized in 100 µl DMSO and agitated on a plate shaker for 10 min. Absorbance was measured at 570 and 690 nm and expressed as relative to vehicle/scramble control as appropriate.

### Animal husbandry

The University of North Carolina at Chapel Hill Institutional Animal Care and Use Committee approved all animal studies and procedures. Female 8-12-week-old C57BL6/NCrl mice (Charles River, Wilmington, MA) were acclimated for one week, then randomized to either a 60 kcal% high-fat diet (Research Diets D12492, New Brunswick, NJ) or an isonutrient-matched control diet with 10 kcal% fat (Research Diets D12450J) for 15–20 weeks to generate control (nonobese) and diet-induced obese (DIO) phenotypes, respectively. Mice were provided ad libitum access to food and water, and were monitored by vivarium staff daily for signs of dehydration, pain, or distress.

### TNBC primary tumor model

Scramble (control) or GPX4-knockdown metM-Wnt^lung^ (5 × 10^4^/50 µl) cells in PBS were orthotopically transplanted into the fourth mammary fat pad of C57BL6/NCrl mice as specified in the experimental designs described below. Body weight was measured weekly, and tumor volume (0.5 × length × width^2^) was measured at least thrice weekly.

#### Immunotherapy treatment

Mice (*n* = 60) were placed on DIO diet for 15 weeks and then were randomized to orthotopic injection with 5 × 10^4^ metM-Wnt^lung^ cells containing either an shRNA construct targeting GPX4 (*n* = 30 mice) or a scramble shRNA construct (*n* = 30 mice). Four days later, mice were randomized to receive an immunotherapy cocktail of anti-PD1 (200 µg/mouse, BioXCell #BE0146) and anti-CTLA4 (100 µg/mouse, BioXCell, #BE0131) (*n* = 30, 15 shGPX4 and 15 shScramble) or an isotype IgG control (300 µg/mouse, BioXCell #BE0090) (*n* = 30, 15 shGPX4 and 15 shScramble) every 3 days via intraperitoneal injection until average tumor size in any treatment group reached a volume of 1500 mm [3]. At this study endpoint, mice were euthanized, and primary tumors were excised and weighed.

#### Chemotherapy treatment

Mice (*n* = 120) were randomized to a DIO diet (*n* = 60) or a control diet (*n* = 60) for 20 weeks and then were randomized to orthotopic injection with 5 × 10^4^ metM-Wnt^lung^ cells containing either an shRNA construct targeting GPX4 (*n* = 60 mice, 30 of each diet) or a scramble shRNA construct (*n* = 60 mice, 30 of each diet). Five days later, mice were randomized to receive carboplatin (50 mg/kg) (*n* = 60 mice, 15 DIO shGPX4, 15 control shGPX4, 15 DIO shScramble, 15 control shScramble) or a vehicle control (*n* = 60 mice, 15 DIO shGPX4, 15 control shGPX4, 15 DIO shScramble, 15 control shScramble), once weekly via intraperitoneal injection until average tumor size in any treatment group reached a volume of 1500 mm [3]. At this study endpoint, mice were euthanized, and primary tumors were excised and weighed.

### TNBC lung colonization model

Nonobese mice (*n* = 5/group) were randomized to receive 10^6^/100 µl control (scramble) or GPX4-suppressed metM-Wnt^lung^ cells via the lateral tail vein. Mice were monitored daily for altered breathing, and at the first indication of labored breathing (which occurred 21 days post injection) all mice were euthanized. The lungs were removed and weighed.

### RNA extraction, cDNA synthesis + qPCR

RNA was isolated using Omega E.Z.N.A. HP Total RNA Kit (BioTek, Winooski, VT), according to manufacturer instructions. cDNA was made with 2 µg RNA and the High-Capacity cDNA Reverse Transcription Kit, according to manufacturer instructions (ThermoFisher Scientific, Waltham, MA). Quantitative PCR was performed using Universal SYBR Green Supermix (Biorad, Hercules, CA) and run on a Viia7 Real-Time PCR system (ThermoFisher Scientific). Primer sequences were obtained from PrimerBank [29] and purchased from IDT (Coralville, IA). *Rpl4*,* Rplp0*, and *Gapdh* were used as housekeeping genes. The relative difference in gene expression level was calculated using the delta-delta-cycle threshold method.

### Gene Set Enrichment Analysis (GSEA)

Affymetrix transcriptomic profiling data of E0771 and metM-Wnt^lung^ tumors from DIO and nonobese (control) mice from previous studies conducted (GSE230471, GSE230470, GSE272326) [30, 31] were aggregated and expression levels generated via Transcriptome Analysis Console (Affymetrix, Santa Clara, CA) to correct for batch effects. GSEA [32] was conducted using the Hallmark gene sets [33]. FDRq < 0.05 was considered significant.

### Western immunoblotting

Western blotting was performed using 50 µg of protein isolated in RIPA buffer supplemented with protease inhibitor cocktail (Sigma Aldrich) and phosphatase inhibitors (sodium orthovanadate, sodium pyrophosphate, and β-glycerophosphate). Protein samples were resolved on 4-17.7% gradient polyacrylamide gels and transferred to nitrocellulose membranes (Bio-Rad). After blocking for 1 h in 5% non-fat milk, membranes were incubated overnight at 4 °C with anti-PC (1:1000, #16588-1-AP, Proteintech, Rosemont, IL), anti-β-actin, (1:5000, #sc-47778, Santa Cruz Biotechnology, Dallas, TX), anti-Nrf2 (1:500, #12721, Cell Signaling Technology, Danvers, MA), or anti-GPX4 (1:000, #144–01933, Raybiotech, Peachtree, GA) antibodies. Membranes were then incubated with secondary IRDye 680 RD goat anti-mouse (LI-COR #926-68070, 1:10,000) or IRDye 800CW goat anti-rabbit (LI-COR #926-32211, 1:10,000) antibody. Antibody binding was detected with the Odyssey Imaging System (LI-COR, Lincoln, NE, USA). Images were analyzed via near-infrared fluorescence using Image Studio Lite software (version 5.2.5, LI-COR Biosciences, Lincoln, NE, USA).

### Flow cytometry

Mammary tumors (*n* = 8/group) were dissociated into single cells using Miltenyi Biotec mouse tumor dissociation kit according to manufacturer’s protocol with enzyme R reduced to 20%. Leukocytes were enriched using a Percoll gradient, which were passed through a 70 μm filter to ensure single cells. Fc receptors were blocked with TruStain FcX (1:25, Biolegend) prior to staining. Cells were stained with live/dead blue viability dye (1:1600) followed by BD Horizon Brilliant stain buffer and antibodies at the following dilutions: 1:75 Ly6G; 1:150 CD19; 1:200 CD11c, CD11b, FoxP3, F4/80, CD4, CD8a, CD3, TIM3, and PD1; 1:300 Ly6C, CD45; and 1:400 CD44. Samples were permeabilized and fixed using Invitrogen FoxP3 Fixation/Permeabilization kit. All samples were measured on a Cytek Aurora cytometer and analyzed using FlowJo Version 10.

### Analysis of published data on GPX4 expression, immunotherapy, and survival in women

We utilized the online database KM Plotter [34] to analyze aggregated gene expression and survival data from GEO, EGA and TCGA for female patients with 9 different cancer types undergoing anti-CTLA4 (*n* = 112), anti-PD-1 (*n* = 411), and anti-PD-L1 (*n* = 459) immunotherapy treatments. Patient data were stratified by high and low tumor GPX4 expression. To determine low and high expression cutoff values, all available cutoff values between the lower and upper quartiles of GPX4 expression were used, and the false discovery rate (FDR) using the Benjamini-Hochberg method was computed to correct for multiple hypothesis testing. The cutoff value with the highest significance (lowest FDR) was determined and used for each GPX4/immunotherapy combination.

### Statistical analyses

Statistical analyses were performed by GraphPad Prism Software Version 10.4.0 (Boston, MA) and statistically significant differences were defined as *p* < 0.05. Statistical tests were two-sided, where applicable. Differences between two groups were analyzed by Student’s t-tests or paired t-tests (when a parametric test was appropriate) or Wilcoxon tests (when a nonparametric paired test was appropriate). Multiple t-tests (parametric or nonparametric) were followed by Holm–Šídák’s multiple comparisons adjustment. Differences between three or more groups were analyzed via one-way or two-way ANOVAs (when a parametric test was appropriate) followed by Tukey’s, Dunnet’s, or Šídák’s multiple comparisons adjustment or Kruskal–Wallis tests (when a nonparametric test was appropriate) followed by Dunn’s multiple comparisons adjustment. Statistical tests are indicated in each corresponding figure legend.

## Results

### GPX4 and PC differentially affect TNBC cell response to redox stress

**Fig. 1:**
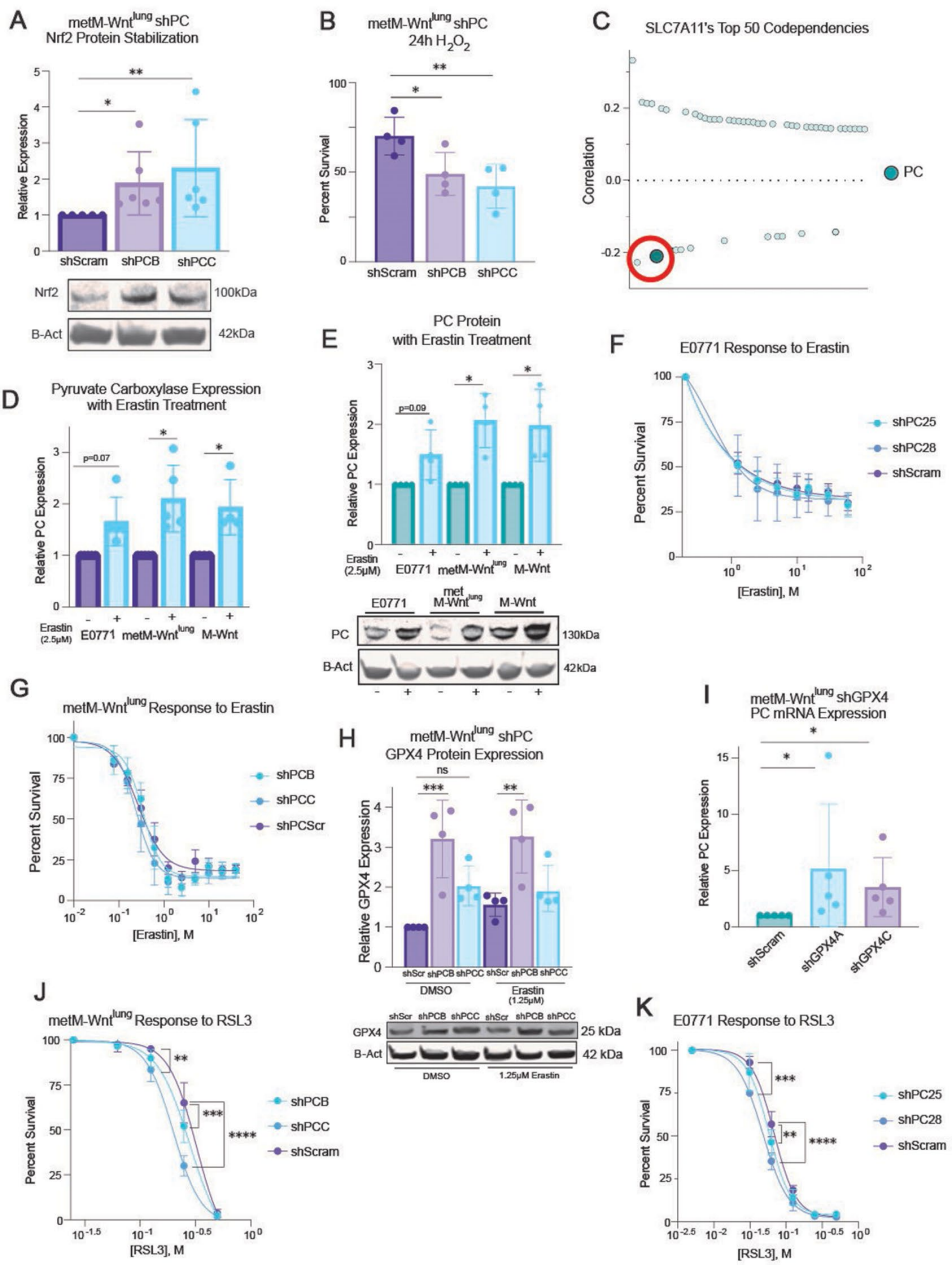
GPX4 and PC differentially affect TNBC cell response to redox stress. (**A**) Immunoblot analysis of control (shScram) and PC-knockdown (shPCB and shPCC) metM-Wnt^lung^ cells probed for Nrf2 and ß-Actin (*n* = 6/group). (**B**) Percent survival of metM-Wnt^lung^ shPC and shScramble cells treated with H_2_O_2_ (500µM) for 24 h (*n* = 4/group). (**C**) Top 50 Codependencies for the gene SLC7A11 from Depmap’s CRISPR dataset graphed by Pearson correlation coefficient. mRNA (**D**) and protein (**E**) expression of PC in E0771, metM-Wnt^lung^, and M-Wnt cell lines treated with erastin (2.5µM) for 24 h. (**D**: *n* = 5/group, E: *n* = 4/group) Percent survival of E0771 (**F**) and metM-Wnt^lung^ (**G**) shPC and shScramble cells treated with erastin for 24 h (**F**: *n* = 5/group, **G**: *n* = 3/group). (**H**) Immunoblot analysis of control and PC-suppressed metM-Wnt^lung^ cells probed for GPX4 and ß-actin after treatment with erastin for 24 h (*n* = 4/group). (**I**) mRNA expression of PC in metM-Wnt^lung^ shGPX4 cells and shScramble controls (*n* = 5/group). Percent survival of metM-Wnt^lung^ (**J**) and E0771 (**K**) shPC and shScramble cells treated with RSL3 for 24 h. (**J**: *n* = 5/group, **K**: 4/group) Statistical significance determined by Kruskal-Wallis test with Dunn’s multiple comparison test (**A, D,I**); one-sample t test (**E**); or two-way ANOVA (**B, F-H, J,K**) with Tukey’s multiple comparison test (**B, G**), Šídák’s multiple comparison test (**H**), or Dunnett’s multiple comparison test (**F, J,K**) (*p < 0.05; **p < 0.01; ***p < 0.001; ****p < 0.0001)

PC knockdown, compared with scramble control, promoted stabilization of the oxidative stress-regulating transcription factor Nrf2 and heightened sensitivity to H_2_O_2_ treatment in the metM-Wnt^lung^ (Fig. 1A-B, Supplementary 1A) and E0771 (Supplementary 1B-C) murine TNBC cell lines. To delineate how PC mRNA expression impacts lipid-specific redox metabolism, we utilized the cancer cell genomics profiling database DepMap [35] (https://depmap.org/portal/) and found that PC was one of the highest codependencies of the cystine/glutamate antiporter and ferroptosis regulator SLC7A11 (Fig. 1C).

Further, in vitro treatment with erastin (a ferroptosis inducer) increased both mRNA and protein expression of PC in TNBC cells (M-Wnt and metM-Wnt^lung^ cells, *p* < 0.05 each, and E0771 cells, p *≤* 0.09; Fig. 1D and E). However, PC knockdown did not alter sensitivity to SLC7A11 inhibition via erastin treatment in either metM-Wnt^lung^ or E0771 cells (Fig. 1F-G). Thus, we hypothesized that a downstream mediator of ferroptosis, such as GPX4, may underpin the heightened sensitivity to redox stress following PC knockdown.

Basal levels of GPX4 protein expression were induced in PC-knockdown metM-Wnt^lung^ and PC-knockdown E0771 cells, compared with scramble controls (Fig. 1H, Supplementary 1D), while PC mRNA expression was induced in GPX4-knockdown metM-Wnt^lung^ cells, compared with its scramble control (Fig. 1I). PC-knockdown metM-Wnt^lung^ and PC-knockdown E0771 cells, as compared to their respective scramble controls, were more sensitive to in vitro GPX4 inhibition via RSL3 treatment (Fig. 1J-K). Thus, we hypothesized that GPX4 may be a targetable determinant of sensitivity to lipid oxidative stress in TNBC.

### TNBC is sensitive to lipid-specific oxidative stress in vitro

**Fig. 2:**
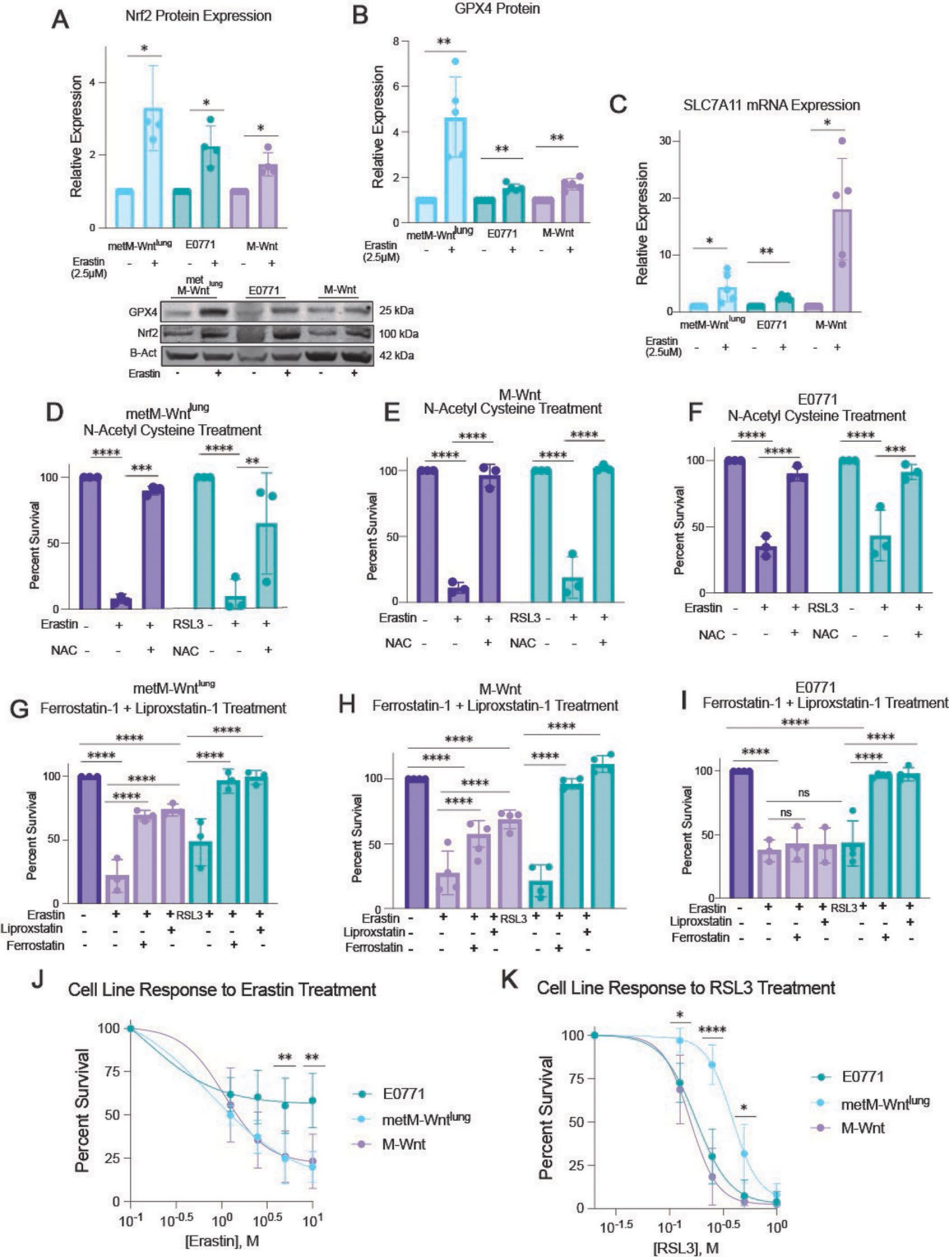
TNBC is sensitive to lipid-specific oxidative stress in vitro. (**A, B**) Immunoblot analysis of metM-Wnt^lung^, E0771 and M-Wnt cells probed for Nrf2 (**A**), GPX4 (**B**) and ß-actin after treatment with erastin for 24 h (A: *n* = 4/group, B: *n* = 5/group). (**C**) mRNA expression of SLC7A11 in metM-Wnt^lung^, E0771 and M-Wnt cells with erastin treatment for 24 h (*n* = 5/group). (**D-F**) Percent survival of metM-Wnt^lung^ (**D**), M-Wnt (**E**) and E0771 (**F**) cells treated with 5µM erastin or 0.5µM RSL3 combined with 500µM N-acetyl cysteine (NAC) for 24 h, relative to a DMSO-treated control for each cell line (*n* = 3/group). (**G-I**) Percent survival of metM-Wnt^lung^ (**G**), M-Wnt (**H**) and E0771 (**I**) cells treated with 5µM erastin or 0.5µM RSL3 combined with 5µM ferrostatin-1 or 5µM liproxstatin-1 for 24 h, relative to a DMSO-treated control for each cell line (*n* = 3–4/group). (**J, K**) Percent survival of metM-Wnt^lung^, M-Wnt and E0771 cells treated with erastin (**J**) or RSL3 (**K**) for 24 h. (*n* = 6/group). Statistical significance determined by Kruskal-Wallis test with Dunn’s multiple comparison test (**A**); one-sample t test (**B, C**); or two-way ANOVA (**D-I**) with Tukey’s multiple comparison test (**D-F, K**), Šídák’s multiple comparison test (**J**), or Dunnett’s multiple comparison test (**G-I**) (*p < 0.05; **p < 0.01; ***p < 0.001; ****p < 0.0001)

Treatment with erastin (an indirect inhibitor of GPX4 via glutathione depletion) increased protein expression of both Nrf2 and GPX4 in M-Wnt, metM-Wnt^lung^, and E0771 cells (Fig. 2A and B). As validation of induction of Nrf2 transcriptional targets, mRNA levels of SLC7A11 were also elevated (Fig. 2C). Direct and indirect GPX4 inhibition, via RSL3 and erastin and treatment respectively, promoted the death of M-Wnt, metM-Wnt^lung^, and E0771 cells (Fig. 2D-F). In each of the cell lines, the global antioxidant N-acetyl cysteine rescued cell viability, consistent with oxidative stress mediating the cytotoxicity (Fig. 2D-F).

Liproxstatin and ferrostatin-1 are radical trapping agents that neutralize lipid peroxides. These agents mitigated the cytotoxic effects of RSL3-induced GPX4 inhibition in each cell line and of erastin-induced GPX4 inhibition in M-Wnt and metM-Wnt^lung^ cells (Fig. 2G-I). In viability assays comparing the 3 cell lines, E0771 cells were the least sensitive to erastin (Fig. 2J, Supplementary 2 A), perhaps related to a greater abundance of genes involved in glutathione metabolism, as seen in comparative analysis of in vivo tumor microarray data (Supplementary 2B). metM-Wnt^lung^ cells proved more resistant than either E0771 or M-Wnt cells to RSL3 (Fig. 2K), aligning with GPX4 protein being highest in this cell line (Supplementary 2 C).

### GPX4 knockdown reduces TNBC metastatic progression and obesity-related tumor burden

**Fig. 3:**
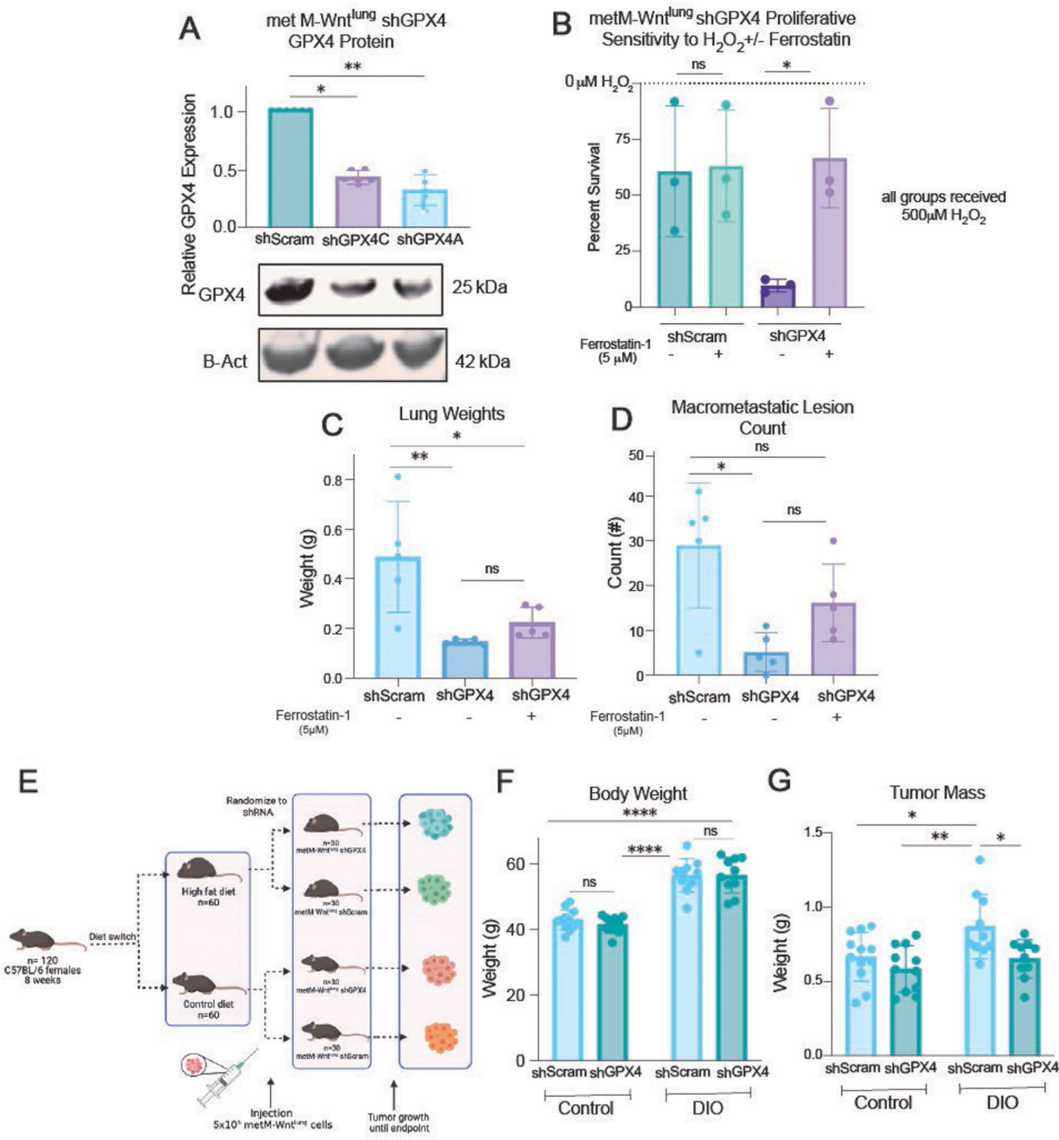
GPX4 knockdown reduces TNBC metastatic progression and obesity-related tumor burden. (**A**) Relative protein expression of GPX4 in shScram and shGPX4 metM-Wnt^lung^ cells (*n* = 5–6/group). (**B**) Percent survival of metM-Wnt^lung^ shGPX4 and shScramble cells treated with 500µM H_2_O_2_ combined with 5µM ferrostatin-1 for 24 h, relative to a DMSO-treated control for each condition (*n* = 3/group). (**C and D**) Murine lung weights (**C**) and pulmonary tumor nodule counts (**D**) 3 weeks post-injection via lateral tail vein with 10^6^ metM-Wnt^lung^ shGPX4 or shScramble cells pre-treated for 48 h with 5µM ferrostatin-1 or a DMSO control (*n* = 5/group). (**E-G**) Experimental timeline (**E**) of metM-Wnt^lung^ shGPX4 or shScramble cells injected into C57BL6/NCrl mice after 20 weeks on either a 60 kcal% high-fat diet (DIO) or 10 kcal% fat control diet (con). Body weights (**F**) and tumor weights (**G**) of mice were taken at study endpoint (*n* = 10–12/group). Statistical significance determined by Kruskal-Wallis test with Dunn’s multiple comparison test (**A, B**); one-way ANOVA with Tukey’s multiple comparisons test (**C, D**); or two-way ANOVA with Tukey’s multiple comparisons test (**F, G**). (*p < 0.05; **p < 0.01; ***p < 0.001; ****p < 0.0001)

Due to the relative insolubility and in vivo instability of RSL3 [36], metM-Wnt^lung^ cells were transduced with shRNA constructs targeting GPX4 to investigate the relationship between GPX4 and TNBC in vivo (Fig. 3A). As expected, GPX4 knockdown increased sensitivity to H_2_O_2_ treatment (Fig. 3B). Similar to what we observed upon treatment with erastin or RSL3 (Fig. 2G-H), the cytotoxic effect of GPX4 depletion was mitigated by ferrostatin-1 (Fig. 3A-B).

GPX4 knockdown reduced lung colonization following tail vein injection of metM-Wnt^lung^ cells in nonobese mice. Acute treatment with ferrostatin-1, as compared with vehicle control, for 24 h prior to injection of GPX4-knockdown metM-Wnt^lung^ cells resulted in a trend toward increased lung weight and macrometastic lesion count although statistical significance was not achieved. The GPX4-knockdown metM-Wnt^lung^ cells, relative to scramble controls, produced lower metastatic tumor burden regardless of ferrostatin-1 pretreatment (Fig. 3C-D).

We next orthotopically injected GPX4-knockdown metM-Wnt^lung^ cells or scramble control cells into the mammary fat pad of nonobese (control) and DIO mice, and monitored tumor growth (Fig. 3E). GPX4 knockdown, compared with scramble, did not produce differences in final body weights (Fig. 3F). However, GPX4 knockdown did reduce tumor burden specifically in DIO but not control mice (Fig. 3G).

### GPX4 knockdown blunts antitumor effects of an ICI treatment regimen in obesity-related TNBC

**Fig. 4:**
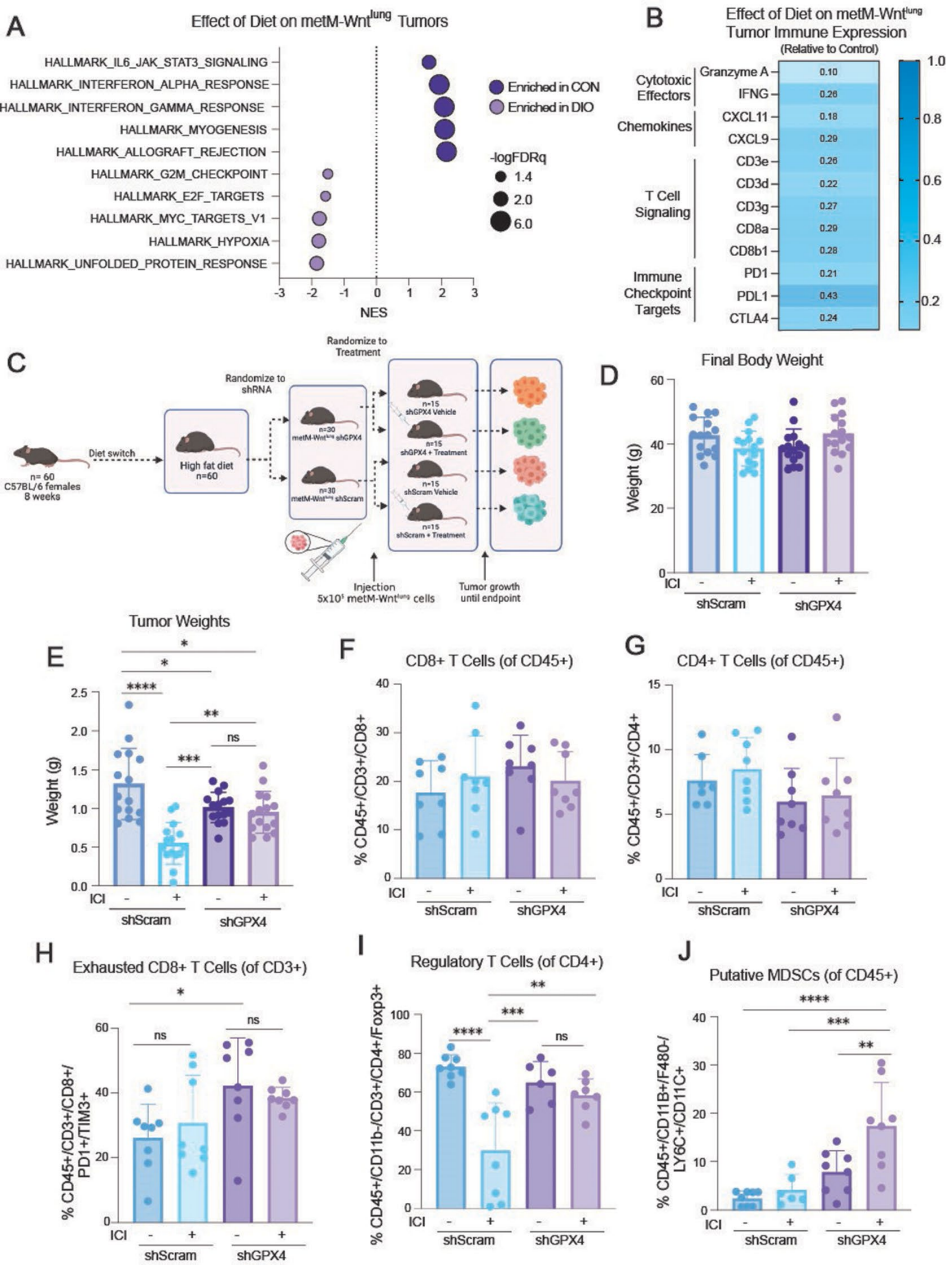
GPX4 knockdown blunts antitumor effects of an ICI treatment regimen in obesity-related TNBC. (**A**) Hallmark gene sets commonly enriched in metM-Wnt^lung^ tumors of control mice vs. DIO mice determined by gene set enrichment analysis (GSEA) (*n* = 12–13/group). (**B**) Heat map of selected immune related genes in metM-Wnt^lung^ tumors from DIO mice, relative to controls (*n* = 13, normalized to gene expression from *n* = 12 tumors from controls) (**C**) Study design to assess the potential of GPX4 expression and immunotherapy treatment to ameliorate metM-Wnt^lung^ tumor growth in a murine model of TNBC and DIO. (**D and E**) Body weights (**D**) and tumor weights (**E**) of DIO mice receiving orthotopic injection of metM-Wnt^lung^ shScramble or shGPX4 cells, combined with either ICI or IgG treatment. Measurements taken at study endpoint (*n* = 15/group). (**F and G**) Percent tumoral CD8+ (**F**) and CD4+ (**G**) cells in tumors, as a percentage of CD45 + cells (*n* = 8/group). (**H**) Percent tumoral PD1+/TIM3 + exhausted CD8 + T cells, as a percentage of CD3 + T cells (*n* = 8/group). (**I**) Percent tumoral FoxP3 + regulatory T cells as a percentage of CD4 + cells (*n* = 8/group). (**J**) Percent tumoral putative MDSCs, as a percentage of CD45 + cells (*n* = 8/group). Statistical significance determined by one-way ANOVA with Tukey’s multiple comparison test (**D**), two-way ANOVA with Tukey’s multiple comparison test (**E-J**) (*p < 0.05; **p < 0.01; ***p < 0.001; ****p < 0.0001)

Obesity impacts the activity and metabolism of immune cells within visceral adipose tissue, driven by changes in hormonal signaling and chronic inflammation [37]. While this impairment in immune function is a mechanism of increased tumor burden in patients with obesity, it is effectively reduced by immunotherapy treatments and indicative of ICI response in several cancer types [38]. To determine the extent to which immunometabolism is altered by body weight phenotype in metM-Wnt^lung^ tumors, we utilized pooled genome-wide transcriptional profiling data of metM-Wnt^lung^ tumors from DIO and control mice analyzed via microarray previously generated by our lab (GSE272326) [30]. By combining these data from a total of 25 tumors over 2 different studies, we performed a comparative analysis of in vivo metM-Wnt^lung^ DIO and control gene expression data utilizing the Hallmark gene sets for GSEA [33]. Of the gene sets most upregulated in the control tumors, or conversely the gene sets most downregulated in the DIO tumors, 4 of the top 5 gene sets were immune-related (Fig. 4A). This confirmed an immunosuppressed phenotype in metM-Wnt^lung^ tumors from DIO mice, which has been previously characterized by a decrease in CD8 + T cell surveillance in this model [30]. In our analysis combining two metM-Wnt^lung^ tumor studies, 8 of the 12 genes most downregulated in tumors from DIO mice were involved in T cell function or activity. Further, metM-Wnt^lung^ DIO tumors had significantly lower transcriptional levels of several immune-regulating genes, particularly those associated with T cell signaling and functionality (Fig. 4B). Thus, the transcripts of several immune-related genes were significantly downregulated in metM-Wnt^lung^ tumors in the context of obesity, concomitant with an immunosuppressive phenotype and posing an opportunity for immunotherapy.

To inform our choice of ICI for testing the effect of GPX4 knockdown plus ICI treatment on TNBC progression in vivo, we used aggregated published human data of tumor GPX4 expression, ICI treatment, and overall survival from female patients in the online resource KMPlotter [34]. Kaplan-Meier analysis revealed that lower GPX4 expression, as determined by the cutoff value with the lowest FDR/highest significance for each ICI target, predicted better survival in women treated with either anti-CTLA4 (Supplementary 3A) or anti-PD1 (Supplementary 3B), yet worse survival in women treated with anti-PDL1 (Supplementary 3C). Hence, we selected a cocktail of anti-PD1 and anti-CTLA4 for evaluation.

DIO mice were randomized to undergo orthotopic transplantation of GPX4-knockdown metM-Wnt^lung^ cells or scramble metM-Wnt^lung^ cells, and then further randomized to receive treatment with the selected ICI cocktail or an IgG isotype control (Fig. 4C). Treatment did not affect final body weights (Fig. 4D). In agreement with our earlier results in DIO mice (Fig. 3G), GPX4 knockdown, as compared with scramble control, reduced tumor burden in IgG control-treated DIO mice (Fig. 4E). ICI treatment, as compared with IgG control, was effective in reducing scramble metM-Wnt^lung^ tumor burden. GPX4 knockdown rendered metM-Wnt^lung^ cells resistant to the ICI treatment (Fig. 4E, Supplementary 3D).

Flow cytometric analysis of tumoral immune cell populations was performed to investigate differential antitumor effects of the ICI treatment by GPX4 expression. Neither GPX4 knockdown nor the ICI regimen shifted relative abundance of the CD4 + or CD8 + T cell populations in the transplanted metM-Wnt^lung^ tumors in DIO mice (Fig. 4F-G). While immunotherapy treatment did not significantly impact the frequencies of exhausted PD1 + TIM3 + CD8 + T cells, suppression of GPX4 increased the relative abundance of PD1 + TIM3 + CD8 + T cells in the IgG-treated group (Fig. 4H).

GPX4 suppression alone had no impact on the relative abundance of Foxp3 + regulatory CD4 + T cell (Tregs) or MDSC populations, but significantly affected these cell frequencies in the context of immunotherapy (Fig. 4I-J). Under the ICI regimen, GPX4 knockdown imparted no change in the Treg population that was markedly reduced by ICI treatment in the scramble tumors (Fig. 4I), and further increased the tumoral MDSC population exclusively in the GPX4 knockdown tumors (Fig. 4J). These GPX4-mediated changes in immunosuppressive cell populations support that GPX4 knockdown promotes an immunosuppressive environment that is associated with an ineffective immunotherapy response.

### GPX4 knockdown synergizes with carboplatin treatment to reduce TNBC burden

**Fig. 5:**
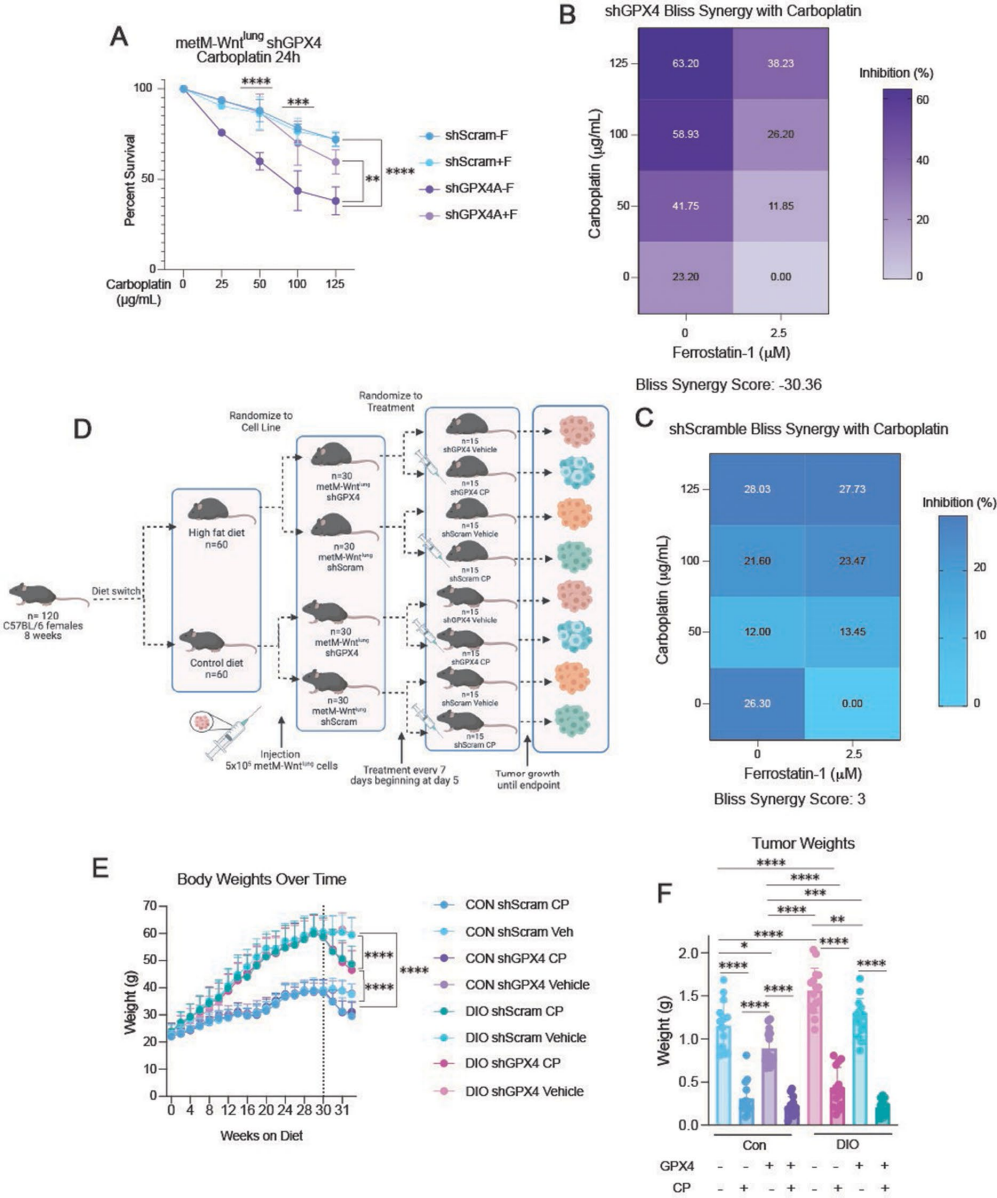
GPX4 knockdown synergizes with carboplatin treatment to reduce TNBC burden. (**A**) Percent survival of metM-Wnt^lung^ shGPX4 and shScramble cells treated with 5µM ferrostatin-1 or a DMSO control combined with carboplatin for 24 h, relative to the shScramble DMSO-treated control treated with carboplatin (*n* = 3–4/group). (**B, C**) Heat map of metM-Wnt^lung^ shGPX4 (**B**) and shScramble (**C**) growth inhibition as determined by BLISS synergy finder utilizing the data from 5 A. (**D**) Study design to assess the potential of GPX4 expression and chemotherapy treatment to ameliorate metM-Wnt^lung^ tumor growth in a murine model of TNBC and DIO. Body weights over time (**E**) and endpoint tumor weights (**F**) of DIO and control mice receiving orthotopic injection of metM-Wnt^lung^ shScramble or shGPX4 cells, combined with either carboplatin or vehicle treatment (*n* = 14–15/group). For the interaction of carboplatin and body weight phenotype, *p* = 0.0002. For the interaction of carboplatin and GPX4 expression, *p* = 0.049. Statistical significance determined by two-way ANOVA with Tukey’s multiple comparison test (**A, E**), or three-way ANOVA with Tukey’s multiple comparison test (**F**) (*p < 0.05; **p < 0.01; ***p < 0.001; ****p < 0.0001)

We sought to determine how GPX4 suppression alters sensitivity to carboplatin treatment in vitro. Inducing lipid oxidative stress by removing ferrostatin-1 treatment from GPX4-knockdown metM-Wnt^lung^ cells increased sensitivity to carboplatin treatment (Fig. 5A). Specifically, the presence of ferrostatin-1 was protective against carboplatin sensitivity in GPX4-knockdown metM-Wnt^lung^ cells (Bliss synergy score, -30.4) yet exerted a negligible effect in scramble metM-Wnt^lung^ cells (Bliss synergy score, 3) (Fig. 5B-C).

Given the strength of the in vitro relationship of GPX4 knockdown and carboplatin sensitivity, we tested the antitumor effects of combining lipid oxidative stress with carboplatin treatment in vivo. Nonobese (control) and DIO mice were randomized to undergo orthotopic transplantation with either GPX4-knockdown metM-Wnt^lung^ cells or scramble control metM-Wnt^lung^ cells, and then further randomized to receive treatment with either carboplatin or vehicle control (Fig. 5D-E). Carboplatin treatment was highly effective at reducing tumor growth regardless of GPX4 or obesity status (*p* < 0.01, each comparison), while GPX4 knockdown modestly reduced tumor progression in both nonobese and DIO mice. Moreover, the interaction of carboplatin plus GPX4 knockdown was significant (*p* = 0.04 Fig. 5F, Supplementary 4A).

## Discussion

By employing preclinical models of TNBC, we demonstrate that defense against lipid oxidative stress is critical in TNBC tumor metabolism and survival through the antioxidant enzyme GPX4. We establish that in TNBC tumors from obese mice, GPX4-mediated redox defense is a targetable vulnerability, and that GPX4 is a powerful regulator of treatment response in our metM-Wnt^lung^ model of TNBC and obesity. Thus, this work demonstrates adaptation to rapid lipid peroxidation as a critical mechanism for obesity-driven TNBC progression and identifies a promising target for therapeutic intervention. Our studies highlight the importance and complexity of alterations in the immune cell populations in the context of diet, disease, and therapeutic intervention. While these findings provide important insights, we acknowledge that the exclusive use of mouse models of TNBC in our studies limits the clinical applicability of our findings. Unfortunately, the immunodeficient murine models required for human cell line or patient-derived xenograft (PDX) studies are resistant to induction of obesity [39]. Thus, given the centrality of obesity to our research question, we conducted all experiments using immune-intact, and diet-induced obesity-sensitive, murine models. We recognize the critical need for future studies to validate these using human-derived cell lines and PDX models to confirm the translational significance of our results, and are developing and testing strategies for utilizing immunodeficient mice for studies of obesity and cancer [40].

Although adipose tissue ROS and reliance on antioxidant activity are known to increase with both breast cancer and obesity [41], and lipid oxidative stress can be utilized to reduce obesity-associated fat accumulation [42], this study is the first to explore the specific interaction between GPX4 inhibition and obesity in breast cancer. Our findings identify GPX4 as a critical regulator of in vivo tumor progression specifically in the context of DIO, delineating a previously unknown relationship between obesity and TNBC metabolism. This work establishes a diet-dependent effect of GPX4 suppression in primary TNBC tumor growth and identifies a promising new anticancer target for breaking obesity-TNBC links.

We demonstrate that efficacy of ICI and dependence on GPX4 are promoted by obesity, but that these approaches behave antagonistically in combination. We and others have shown obesity to blunt antitumor immunity in TNBC models [30, 43]. CD8 + T cells are present at much lower frequencies in TNBC tumors from DIO mice [44], and T cells from patients with obesity and TNBC express greater levels of immune checkpoint genes [45]. Obesity is an important regulator of ICI response in some cancers [46, 47], yet how obesity contributes to ICI response in TNBC is unknown. GPX4 expression in tumors correlates with lower response to immunotherapy treatments (Supplementary 3 A-B), and GPX4 inhibition synergizes with immune checkpoint inhibitors to reduce tumor burden in models of TNBC [48] without obesity.

Myeloid-derived suppressor cells (MDSCs), protumorigenic immune cells that inhibit T cell activity, are increased in number and activity with obesity [49]. In our study, suppression of GPX4 heightened MDSC levels above that of obesity alone. Tumors undergoing lipid oxidative stress release immunogenic cell death signals such as high mobility group box 1 (HMGB1), a mechanism critical for GPX4-mediated tumor targeting [50]. Immunogenic cell death signaling can also result in greater MDSC recruitment to the tumor microenvironment, as has been demonstrated in a GPX4-suppressed murine model of hepatocellular carcinoma [51].

Tumor-specific GPX4 inhibition synergizes with anti-PD1 immunotherapy in murine models of pancreatic, colon and liver cancer [50, 51]. T cell exhaustion has not been associated with tumoral GPX4 expression, but as our DIO study indicated greater numbers of PD1+/TIM3 + CD8 + T cells in shGPX4 tumors, this finding could be a diet-specific effect. Tregs blunt immunotherapy response in TNBC [52] and this cell population was not reduced by ICI treatment in GPX4 suppressed tumors. As immunogenic cell death signals have been shown to reduce intratumoral Treg populations [53], further investigation into the relationship between tumor GPX4 expression, anti-PD-1/CTLA-4 treatment, anti PD-L1/other ICI regimens, and Treg function is warranted.

Platinum-containing chemotherapies induce cell death in part by depleting glutathione and inactivating glutathione peroxidase [21], explaining the synergy between carboplatin and GPX4-suppressed cells in vitro and in vivo. Combining carboplatin with the ROS-inducing compound sulfasalazine reduces the carboplatin IC_50_ in TNBC [54], further supporting the therapeutic strategy of glutathione depletion to enhance chemotherapy efficacy [19]. The statistical significance of the interaction between GPX4 and carboplatin, despite the immense effect of carboplatin we saw in our in vivo study, speaks to the strength of this relationship. Our findings indicate this interaction could be a valuable strategy to target therapeutic resistance in the context of obesity.

In exploring metabolic parameters by which lipid redox homeostasis can be manipulated, we uncovered an interesting and novel relationship between GPX4 (and its upstream regulator SLC7A11) with PC. Our findings suggest that in TNBC, PC is involved in defense not just against oxidative stress, but also lipid-specific oxidative stress that is critical for metabolism and survival. The discovery of this interaction is a novel contribution to the field, and highlights PC as a metabolic target that may be important to therapeutic response in TNBC. Further investigation is warranted as the biochemical and redox underpinnings of cancer biology become increasingly intricate and intertwined.

As the specific interaction between lipid oxidative stress and obesity has not yet been explored in breast cancer, these findings introduce a body weight phenotype-dependent vulnerability in TNBC that impacts efficacy of multiple first line therapies. Our studies highlight the importance and complexity of alterations in the immune cell populations in the context of body weight phenotype, disease, and therapeutic intervention, and can be used to inform future studies at this intersection. We conclude that GPX4-mediated redox defense, alone or in combination with therapies such as carboplatin, is an actionable target for treatment of TNBC, particularly in patients with obesity.

## Electronic supplementary material

Below is the link to the electronic supplementary material.

Supplementary Material 1

## Data Availability

The datasets used and/or analysed during the current study are available from the corresponding author on reasonable request.
