# Supplementary material for: Glutathione peroxidase 4 (GPX4) and obesity interact to impact tumor progression and treatment response in triple negative breast cancer
Source: Cancer Metab. 2025 Feb 25;13:11. doi: 10.1186/s40170-025-00380-8 (PMC11863593; doi:10.1186/s40170-025-00380-8)
Supplement: Supplementary file 1 — Supplementary Material 1 [file 40170_2025_380_MOESM1_ESM.docx]

**
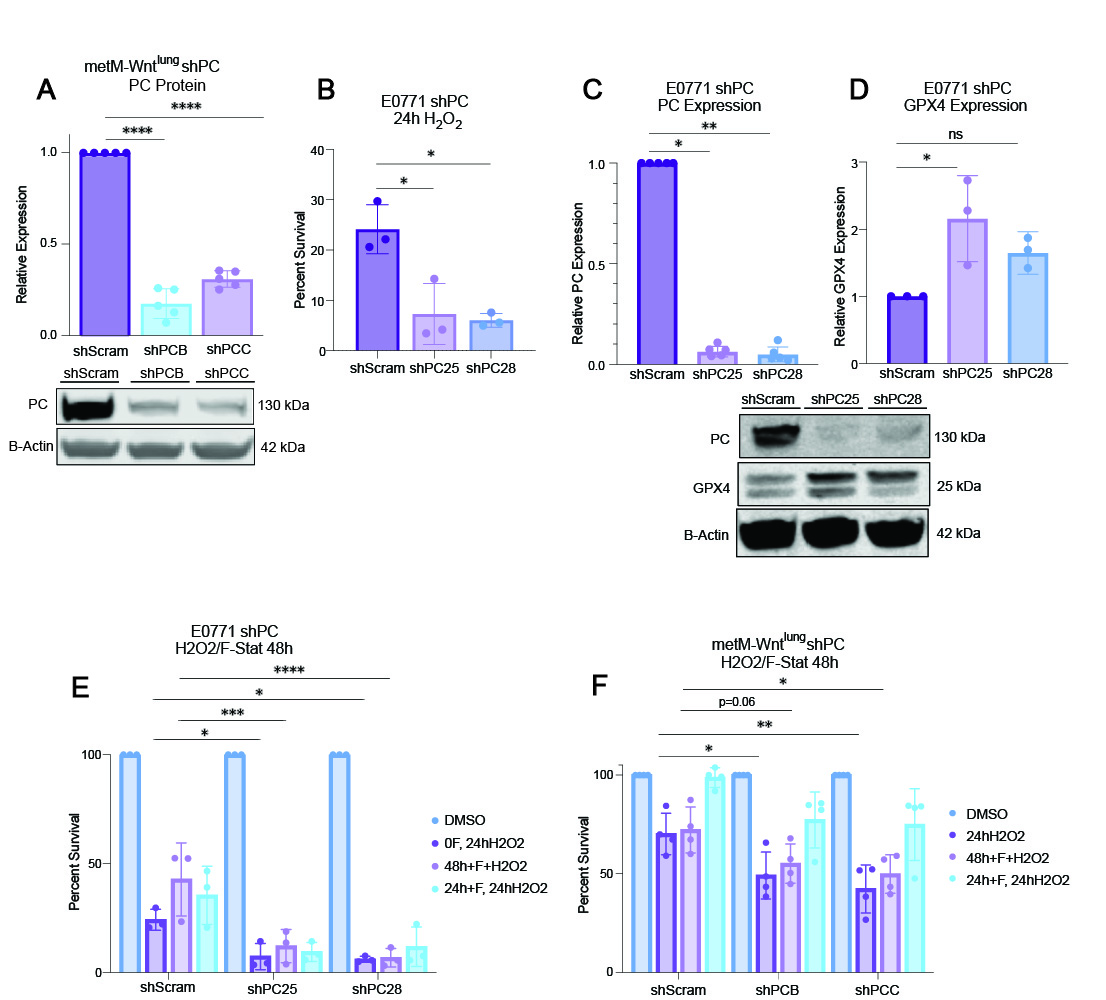
**

**Figure S1: GPX4 and PC differentially affect cell response to redox stress in multiple murine models of TNBC**

(A) Immunoblot analysis of control (shScram) and PC-suppressed (shPCB and shPCC) metM-Wnt^lung^ cells probed for PC and ß-actin (n=5/group). (B) Percent survival of E0771 shPC and shScramble cells treated with H_2_O_2_ (500μM) for 24 h (n=3/group). (C,D) Immunoblot analysis of control (shScram) and PC-suppressed (shPC25 and shPC28) E0771 cells probed for PC (C), GPX4 (D) and ß-actin (n=3-5/group). (E, F).Percent survival of E0771 (E) and metM-Wnt^lung^ (F) shPC and shScramble cells treated with H_2_O_2_ (500μM), 48h of ferrostatin-1 (2.5μM) with H_2_O_2_, or 24h of ferrostatin-1 followed by 24h of H_2_O_2_ (n=3-4/group). Statistical significance determined by one sample t test (A), two-way ANOVA with Tukey’s multiple comparison test (B,E,F), or Kruskal-Wallis test with Dunn’s multiple comparison test (C,D) (*p<0.05; **p<0.01; ***p<0.001).

**
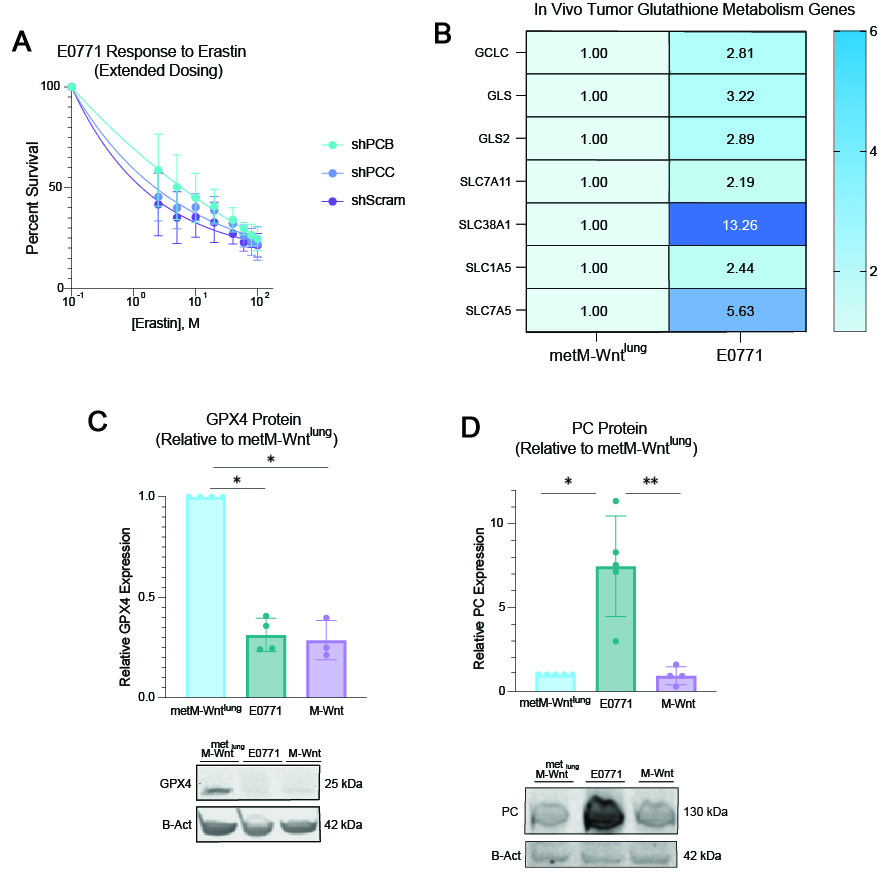
**

**Figure S2: Heterogeneity in TNBC could contribute to ferroptosis sensitivity**

(A) Percent survival of E0771 shPC and shScramble cells treated with erastin for 24 h. (n=4/group). (B) Heat map of selected glutathione metabolism-related genes in E0771 tumors, relative to metM-Wnt^lung^ tumors (n=48, normalized to gene expression from n=25 metM-Wnt^lung^ tumors). (C,D) Immunoblot analysis of metM-Wnt^lung^, E0771 and M-Wnt cells probed for GPX4 (C), PC (D) and ß-actin, normalized to metM-Wnt^lung^ expression (n=3-5/group). Statistical significance determined by two-way ANOVA with Šídák’s multiple comparison test (A), Kruskal-Wallis test with Dunn’s multiple comparison test (C,D) (*p<0.05; **p<0.01; ***p<0.001).

**
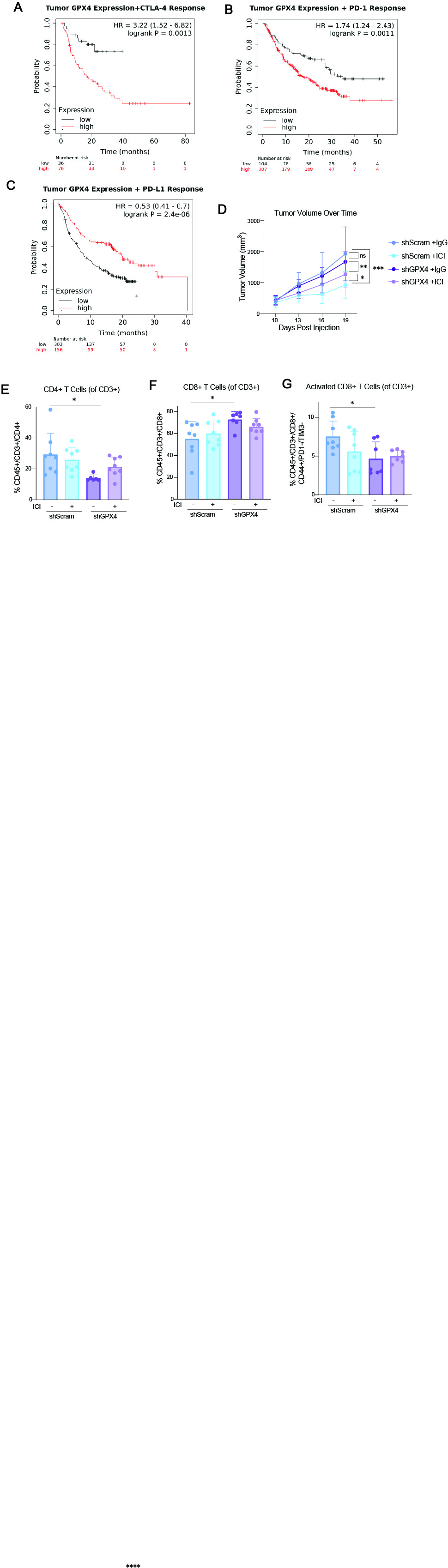
**

**Figure S3: Tumoral GPX4 expression impacts ICI response in human and murine models**

(A-C) Kaplan Meier plots^34^ for female patients with cancer undergoing anti-CTLA4 (A) (n=112), anti-PD-1 (B) (n=411) and anti-PD-L1 (C) (n=459) immunotherapy treatments, stratified by high and low tumor GPX4 expression. (D) Tumor volumes of DIO mice receiving orthotopic injection of metM-Wnt^lung^ shScramble or shGPX4 cells, combined with either ICI or IgG treatment (n=15/group). (E) Percent tumoral CD4+ T cells, as a percentage of CD3+ T cells (n=8/group). (F) Percent tumoral CD8+ T cells, as a percentage of CD3+ T cells (n=8/group). (G) Percent tumoral CD44+ CD8+ T cells, as a percentage of CD3+ T cells (n=8/group). Statistical significance determined by logrank P value and hazard ratio with 95% confidence intervals (A-C), two-way ANOVA with Fisher’s LSD (D), and two-way ANOVA with Tukey’s multiple comparison test (E-G) (*p<0.05; **p<0.01; ***p<0.001).


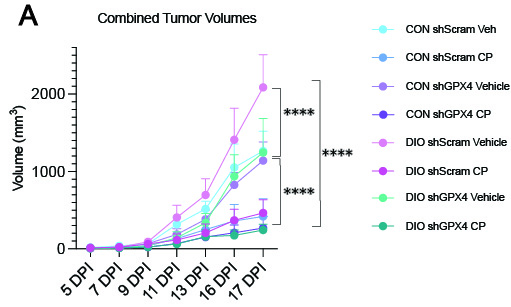


**Figure S4: Carboplatin tumor volumes**

(A) Tumor volumes of DIO and control mice receiving orthotopic injection of metM-Wnt^lung^ shScramble or shGPX4 cells, combined with either carboplatin or vehicle treatment (n=14-15/group). Statistical significance determined by two-way ANOVA with Tukey’s multiple comparison test (****p<0.0001).
